# Introducing the Special Issue on Label-Free Quantitative Phase Imaging Honoring Prof. Gabriel Popescu, a Pioneer in Biomedical Optics

**DOI:** 10.1117/1.JBO.29.S2.S22701

**Published:** 2024-12-19

**Authors:** Stephen A. Boppart, Catherine A. Best-Popescu, Randy A. Bartels, Kevin Eliceiri

**Affiliations:** a University of Illinois Urbana-Champaign, Grainger College of Engineering, Urbana, Illinois, United States; b Morgridge Institute for Research, Madison, Wisconsin, United States; c University of Wisconsin – Madison, College of Engineering, Madison, Wisconsin, United States

## Abstract

The editorial introduces the JBO special issue honoring Prof. Gabriel Popescu, Pioneer in Biomedical Optics.

Prof. Gabriel Popescu was a renowned optical scientist, engineer, and international thought-leader in the field of label-free quantitative phase imaging (QPI) in biophotonics and biomedical optics. His pioneering work established a new field and focus on QPI for our research community, and his unbridled spirit and passion for excellence in his research and in developing collaborative relationships around the world led to a broad sense of partnership and cohesiveness that moved the field and all of us forward.

Prof. Popescu’s seminal papers included some of the first for using QPI to detect subtle nanometer-scale fluctuations in the membranes of cells, including changes in red blood cells associated with sickle cell anemia, malaria, and other hematologic disorders. As an exquisitely sensitive measurement platform, Prof. Popescu applied QPI for measuring the dry mass of bacteria and eukaryotic cells and associated dynamic changes in neurons with the movement of organelles and the shuttling of intracellular vesicular packages. QPI changes between healthy and diseased cells and tissues heralded diagnostic opportunities that continue to be investigated today by groups around the world. Extensions of QPI led to new techniques such as Spatial Light Interference Microscopy (SLIM) and Gradient Light Interference Microscopy (GLIM) for not only 2D image generation but also 3D volumetric imaging of cells, tissues, embryos, spheroids, and organoids. All of these advances promoted a label-free approach that avoided the perturbative influences of dyes, stains, or nanoparticles to yield a more natural biological state, and would facilitate more rapid translation into clinical use.

Prof. Popescu was incredibly prolific in his enthusiastic and stimulating pursuit of QPI and label-free optical imaging approaches. He published over 200 peer-reviewed publications in some of the top biophotonics and biomedical optics journals in our field, and the wide applicability of his QPI techniques led to publications in broader scientific and clinical journals. He had over 30 patents related to his work, and founded Phi-Optics, Inc. that helped commercialize and disseminate his innovations to a larger group of users in our field. He was an inspiring and supporting mentor to many graduate students, post-docs, and research scientists, and established a biophotonics summer school series along with foundational multi-volume textbooks to inform, educate, and train future generations of scientists, engineers, and clinicians on the potential and power of QPI. His national, professional, and institutional service contributions as a thought-leader reflected his high degree of investment and engagement in our community that will be missed.

In this special issue, we wish to highlight the extensive review of QPI and Prof. Popescu’s work by Goswami et al. (part 1, doi 10.1117/1.JBO.29.S2.S22713; part 2, doi 10.1117/1.JBO.29.S2.S22714). Dr. Goswami was one of Prof. Popescu’s last postdoctoral research fellows, and this review by her and Prof. Anastasio, a faculty colleague and collaborator, seeks to capture the broadest extent of application and impact of QPI. Numerous other papers in this special issue feature the application of label-free QPI, including methods for capturing heterogeneous and dynamic biological systems (Skala et al., doi 10.1117/1.JBO.29.S2.S22702), for 3D cell segmentation (Barman et al., doi 10.1117/1.JBO.29.S2.S22705), and for improving the diagnostic precision in cancer medicine (Liu and Uttam, doi 10.1117/1.JBO.29.S2.S22705). These represent only a very small subset of the work by and inspired by Prof. Popescu over the 20 years of his shortened academic career.

Prof. Popescu’s many collaborative relationships and projects coalesced into his efforts for a new NIH/NIBIB Technology Resource (P41) and Center for Label-free Imaging and Multi-scale Biophotonics (CLIMB) that was funded and launched shortly after his tragic and unexpected passing in 2022. CLIMB represents Prof. Popescu’s vision and legacy that will serve as an international resource for label-free optical imaging, and its applications for not only basic biological discovery, but also clinical translation and impact.

As guest editors for this special issue, we are therefore pleased to share the 16 papers in this issue. The breadth of these papers reflects the applicability, significance, and impact of the work that was related to and/or inspired by the work of Prof. Popescu. These survey the field and include:

• Label-free microscopy of cells or tissues
• Current and potential applications of quantitative phase imaging
• Established or emerging label-free imaging technologies
• Clinical opportunities of label-free imaging
• Computational challenges or achievements in label-free imaging
• Label-free multiscale imaging opportunities or achievements
• Future directions in label-free imaging

Collectively, the authors who contributed papers to this special issue have demonstrated how an inspirational visionary in our field can bring about new ideas and innovations, new technologies and techniques, new applications and discoveries, and new methods and measurements that can both reveal scholarly excellence to promote our field, as well as have a translational clinical impact to improve our lives. The guest editors would like to thank JBO Editor-in-Chief Brian Pogue, and the JBO support staff, as well as the contributors, associate editors, and reviewers, for their invaluable dedication and support of this special issue, and for this opportunity to recognize and honor the late Gabriel Popescu as a true pioneer in our field.

